# Psychiatry: Peter Falkai

**DOI:** 10.1007/s00702-022-02491-8

**Published:** 2022-04-07

**Authors:** Peter Falkai

**Affiliations:** Department of Psychiatry, University of Munich Hospital, Nussbaumstrasse 7, 80336 Munich, Germany

Prof. Riederer has not only worked in the field of neurodegenerative disorders, covered in the first part of the book, but also in the field of psychiatric disorders. The seven chapters of the psychiatry section deal with various aspects of schizophrenia, depression, autism and other topics, which amongst others have been main aspects of Peter Riederer’s scientific research interests. The first chapter by Peter Falkai and colleagues describes the neurodevelopmental hypothesis of schizophrenia, introduced more than 30 years ago. They state that schizophrenia is a consequence of failed neurodevelopmental processes leading to a dysfunctional neuronal network forming the basis for a psychosis proneness, and that subsequently, significant research efforts were made to prove the neurodevelopmental or the neurodegenerative perspective. Key arguments speaking for or against the two hypotheses leading to a concept with both aspects positioned side by side are summarised in this chapter, which is dedicated to the 80th birthday of Peter Riederer, who has and still is contributing significantly with his research to understanding the pathophysiology of severe mental and neurological disorders. He has fostered generations of researchers to follow him, for which all of us are grateful.

In the second chapter, Tanja Michel discusses the superoxide dismutase isozymes in cerebral organoids from autism-spectrum disorder patients. The aim of this study was to measure superoxide dismutase 1–3 in autistic cerebral organoids as an in vitro model of human foetal neurodevelopment. However, cerebral organoids recapitulate many aspects of human neurodevelopment, but the diffusion restriction may render efforts in modelling differences in oxidative stress futile due to the intrinsic hypoxia and central necrosis.

In the third chapter of this psychiatry section, Gavin Reynolds provides a brief overview of one trajectory of his research into the neurobiology of schizophrenia that started in the Riederer lab in Vienna investigating dopamine and the D2 receptor. Subsequent research to understand findings of increased dopamine resulted in the identification of reduced GABAergic innervation, culminating in the finding of a deficit in the parvalbumin-containing subtype of GABAergic neurons. Most recent work has been studying how changes in DNA methylation of the parvalbumin gene may relate to these findings in psychotic illness and its animal models.

Investigating mechanisms of cognitive control training: neural signatures of PASAT performance in depressed patients is the topic of the fourth chapter of this section, discussed by Anja Sommer, Andreas Fallgatter and Christian Plewnia. They found significantly larger amplitudes after negative than positive feedback for the P300 and late positive potential (LPP). However, this difference was not significant for the feedback-related negativity (FRN). Moreover, no associations of valence-specific ERPs and PASAT performance nor depressive symptoms were found. This indicates that depressed patients seem unable to use neural activation in late feedback processing stages (P300, LPP) to adapt accordingly. Moreover, lack of valence-specific neural reaction in early feedback processing stages (FRN) might point towards emotional indifference in depressed patients.

In the fifth chapter, Heike Weber and colleagues from the Deckert group deal with the association of Polygenic Risk Scores, traumatic life events and coping strategies with war-related PTSD diagnosis and symptom severity in the South Eastern Europe (SEE)-PTSD cohort. They conclude that the present PRS application in the SEE-PTSD cohort confirms modest but significant polygenic risk for PTSD diagnosis. Environmental factors, mainly the intensity of traumatic life events and negative coping strategies, yielded associations with PTSD both categorically and dimensionally with *p* values that are more significant. This suggests that, at least in the present cohort of war-related trauma, the association of environmental factors and current individual coping strategies with PTSD psychopathology was stronger than the polygenic risk.

The sixth chapter of the psychiatry section deals with the therapeutic drug monitoring in children and adolescents with schizophrenia and other psychotic disorders using risperidone. Karin Egberts and colleagues discuss that knowledge on dose–concentration relationships of risperidone treatment in children and adolescents with schizophrenia or other psychotic disorders is, however, scarce and no age-specific therapeutic ranges have been established yet. Thus, the preliminary data of their study may contribute to the definition of a therapeutic window in children and adolescents with schizophrenic disorders treated with risperidone, and that TDM is recommended in this vulnerable population to prevent concentration-related adverse drug reactions.

In the seventh and last chapter of the psychiatry section, Miriam Bieber and colleagues deal with a randomized controlled clinical trial on the feasibility and effects of 12 weeks yoga and aerobic exercise training in patients with major depressive disorder. They found that high dropout rates in the more self-centred Yoga intervention and the control group may have been caused by a lack of motivation and social support and may have biased the effects of their method. However, they suggest in future studies to implement familiar movement patterns beside the new and complex patterns may help to increase the motivation of the patients and may reduce dropout rates.

Peter Falkai’s personal dedication to Peter Riederer:

Peter Riederer has contributed important input in the field of psychiatry, and he has thus promoted and encouraged the significance of multi-transmitter deficits in depression and the dopaminergic receptors for schizophrenia. His works in the field of the signal transduction cascade in addiction disorders are none the least noteworthy.

Apart from his scientific excellence and his sense for important research topics Peter Riederer has also ever had an instinct for talented junior scientists. Beside his scientific creativity, his commitment, his contentiousness and his diligence, Peter Riederer has also ever practiced a very human and social interaction towards his colleagues. During his professorship in Vienna, he is said to have enjoyed hosting doctoral students in the famous Cafe Landtmann, to join forces on serious and important scientific topics in a relaxed and casual atmosphere.

